# Projection of climate change impact on the occurrence of drought events in Poland

**DOI:** 10.1038/s41598-025-90488-0

**Published:** 2025-02-15

**Authors:** Babak Ghazi, Hossein Salehi, Rajmund Przybylak, Aleksandra Pospieszyńska

**Affiliations:** 1 Department of Meteorology and Climatology, Faculty of Earth Sciences and Spatial Management, Nicolaus Copernicus University, Toruń, Poland; 2 Department of Physics, University of Trento, 38123 Trento, Italy; 3 Centre for Climate Change Research, Nicolaus Copernicus University, Toruń, Poland

**Keywords:** Climate change, Droughts, SPEI, CMIP6, Poland, Climate change, Projection and prediction

## Abstract

In the era of human-induced climate change, droughts are one of the extreme events that can severely impact water resources, regional agriculture and the ecological environment. A reliable projection of droughts for the future is crucial with the rapid global increase in the frequency and intensity of droughts. This research aims to project the future of droughts in Poland by the end of the 21st century. To achieve this, we use a multi-model mean ensemble of 26 downscaled and bias-corrected high-resolution general circulation models (GCMs) under Shared Socioeconomic Pathways (SSP1–2.6, SSP2–4.5, SSP3–7.0, and SSP5–8.5) scenarios from Coupled Model Intercomparison Project Phase 6 (CMIP6) to project changes in temperature and precipitation. Then, estimation of droughts was completed for the periods 2031–2060 and 2071–2100 according to the Standardized Precipitation Evapotranspiration Index (SPEI) at three-time scales of 1, 6 and 12 months to address three types of droughts (i.e., meteorological, agricultural and hydrological, respectively). The temperature and precipitation projection revealed that temperature will increase in both future periods, which is dramatic under the SSP3–7.0 and SSP5–8.5 scenarios. However, precipitation fluctuates based on scenarios with a slight increase, excluding far-future periods under SSP3–7.0, and SSP5–8.5 scenarios. The projected changes in droughts based on SPEI under SSP scenarios showed a decrease in frequency in the near-future, but an increase under SSP2–4.5, SSP3–7.0 and SSP5–8.5 in the far-future. The number of severe and moderate droughts is expected to rise, with a notable increase in agricultural and hydrological droughts (SPEI-6 and SPEI-12) under SSP3–7.0 and SSP5–8.5.

**Supplementary Information:**

The online version contains supplementary material available at 10.1038/s41598-025-90488-0.

## Introduction

Global warming is among the greatest environmental challenges of our time. Climate change and extreme events will significantly affect many ecosystems and various aspects of people’s lives, including their health^1,2^. Global mean temperatures will continue to increase by the end of the century under Shared Socioeconomic Pathways (SSP) scenarios, according to the *Sixth Assessment Report on Climate Change* by the Intergovernmental Panel on Climate Change^2^. Scientific research shows that modern global warming largely influences atmospheric precipitation and air temperature and will increase occurrences of extreme events like droughts and floods^3–5^. Thus, it is extremely important to study the risk of the occurrences of extreme events in the future^6,7^. Drought is an important and complex sub-seasonal event that has a major effect on various socioeconomic activities, agriculture, energy production and water resources^8,9^. Droughts occur at broader spatiotemporal scales than other extreme events and frequently last for longer periods of months to years^10,11^. In recent years, many studies have projected that the spatial extent, severity and frequency of the various types of droughts will increase globally^12–15^. Europe is one of the regions of the world that has experienced various droughts recently, and there is a high drought risk in the future^12,16^. For example, Poland has experienced several extreme and severe droughts in recent years, such as in 2018–2019 and spring of 2020 and destructive droughts in 1992, 1994, 2006, 2008 and 2015 ^17–19^. In some regions of Poland, crop farms affected by drought have lost more than 70% of the value of their crops^20^. Understanding and providing a picture of the future of this complex phenomenon can provide valuable information in the adaptation and possible measurements to deal with it. Therefore, an assessment of climate change impacts on the future of droughts in this region seems urgently needed.

In recent decades, the impact of climate change in Poland has been the subject of research by many scientists under representative concentration pathway (RCP) and shared socioeconomic pathways (SSP) scenarios from Coupled Model Intercomparison Project Phase 5 and 6 (CMIP5, CMIP6, respectively)^17,21–26^. However, studies focusing on the impact of climate change on the occurrence of drought in Poland in the future are limited. For example, Meresa, et al.^21^ assessed droughts under RCP scenarios from CMIP5 for only ten catchments in Poland, which covers only small regions of Poland. Osuch, et al.^27^ evaluated the trends of projected droughts in Poland based on the standardized precipitation index (SPI) under the A1B scenario from the oldest version of GCM models from CMIP3. Also, the study does not involve the impact of temperature on droughts in the study area. In addition to the mentioned limitations in previous studies, which were focused on case studies in some parts of Poland, there is no research on the projection of droughts using novel CMIP6 simulation models and involving temperature as the dominant variable in the occurrence of droughts. On the other hand, to the author’s best knowledge, almost no study evaluated the impact of climate change on the occurrence of drought events in Poland based on datasets from CMIP6 under SSP scenarios. Thus, this research’s main objective is to evaluate the expected influence of climate change on drought occurrences in Poland by the end of the 21st century. The most important advantage and state-of-the-art contribution of this study is an assessment of droughts for entire regions of Poland and projection of frequency and intensity of all types of droughts (i.e., meteorological, agricultural and hydrological) in Poland by the end of 21st century under the latest GCM models and scenarios from CMIP6.

The primary aims of this research are as follows:

(i) To investigate variations in the main climatic variables (i.e. temperature, precipitation) in Poland in future periods under climate change scenarios from CMIP6,
(ii) To evaluate the impact of changes on the frequency and severity of droughts in a future period in Poland,
(iii) Assessment of various types of droughts (i.e., meteorological, agricultural and hydrological) in Poland.

The results of this research will help policymakers in Poland to develop scenarios and robust climate change adaptation strategies. In addition, the output of the study will contribute to improving research on the future of droughts in Europe, particularly in Central Europe.

## Materials and methods

**Fig. 1:**
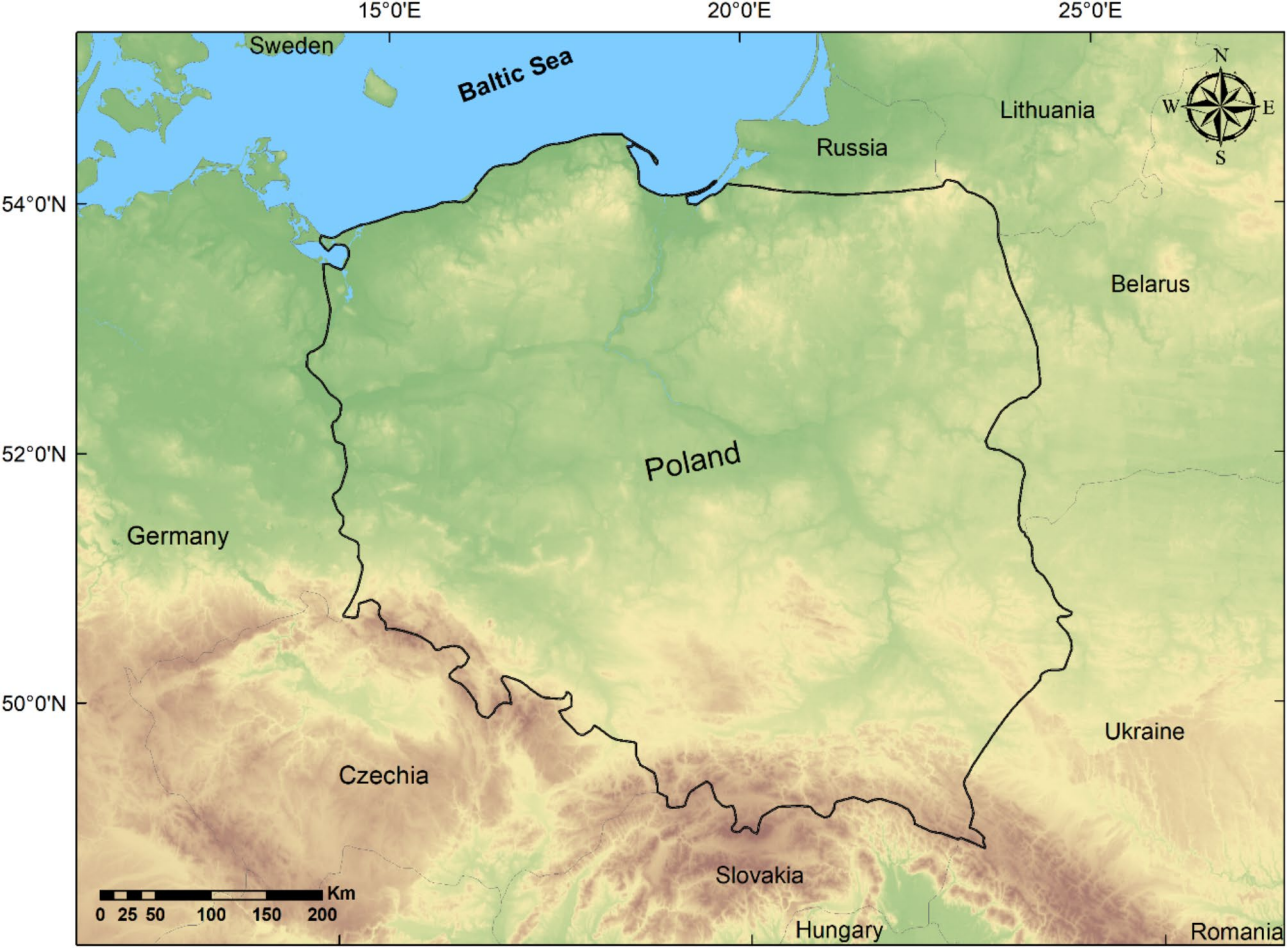
Geographical location of Poland created using ArcMap 10.8.2 (http://www.esri.com/).

In this study, we assess the future of drought occurrences under climate change in the region of Poland in Central Europe (Fig. 1). The impact of air masses from the Atlantic Ocean and the Eurasian landmass characterizes Poland’s climate. The greater part of Poland is classified as Dfb (cold, no dry season, warm summer) according to the Köppen–Geiger climate classification^28^. Poland’s average annual temperature from 1951 to 2008 was 7.9 °C, and annual precipitation was 623 mm from 1961 to 2009. In addition, the temperature spatially ranges from 6 to 10 °C in the northeast and southwest of the region, respectively. Moreover, the precipitation totals in central parts and the mountain area range between 800 and 1,100 mm^29,30^.

GCMs are among the most advanced and robust tools for projecting climate variable changes for a future period. Despite their capability to evaluate the impact of climate change, there are uncertainties and limitations in these models. To overcome and address these uncertainties and limitations in individual models, many studies recommended that a combination of several GCMs be employed to assess changes in the future of climate variables and extreme events^31–34^. Thus, this research, to increase the robustness of projection, used a multi-model ensemble of 26 GCMs (Table S1) from CMIP6. The selection of models was based on the availability of variables and scenarios.

The applied GCMs are a set of high-resolution, downscaled and bias-corrected models from CMIP6 models output, namely NASA Earth Exchange Global Daily Downscaled Projections (NEX-GDDP)^35^. Analyses were carried out under four SSP scenarios: SSP1–2.6, SSP 2–4.5, SSP3–7.0 and SSP 5–8.5. The details regarding each scenario are available in reference research^36^. The effectiveness of the NEX-GDDP dataset in the assessment of extreme events (including droughts) under climate change has been proved in previous studies^37–43^.

In the selected GCMs, three 30-year long periods have been used for analysis: a historical period (1985–2014) as a reference period, a near-future period (2031–2060) and far-future period (2071–2100).

### Drought indices

Assessment and identification of droughts are conducted based on various indices. In drought evaluation, researchers employ single or multiple indices based on the availability of datasets and the scope of research works. We used the Standardized Precipitation-Evapotranspiration Index (SPEI) to assess droughts under climate change scenarios in Poland. SPEI was developed based on the Standardized Precipitation Index (SPI)^44^ and is one of the most widely used indices in quantifying droughts.

Since the SPI index depends on precipitation, it has a limitation in detecting droughts caused by temperature^45^. For example, Przybylak, et al.^46^ evaluated drought occurrences in Poland for a long historical period using SPI only. The authors concluded that SPI, which used only precipitation data, could not capture the recent droughts in Poland, which are mainly formed because of rising temperatures. An increase in temperature will cause water loss through evaporation^47^. Thus, indices combining temperature and precipitation have shown better results in detecting various types of droughts^48,49^. SPEI is based on differences between potential evapotranspiration and precipitation, and temperature is also considered in drought calculation^50^. The SPEI is a standardized index that does not need to be calibrated and it can be calculated on various timescales. Also, many studies confirmed that the SPEI has shown better capability in assessing droughts under climate change^51–53^.

In general, droughts are classified based on frequency, duration and intensity^54,55^. If the meteorological conditions persist for a short duration (i.e., a few weeks), a drought is classified as a “meteorological drought”. If dry conditions last for 3–9 months, then, due to the impact on soil moisture, a drought is classified as an “agricultural drought”^56^. Dry conditions lasting more than 9 months, due to the impact on the hydrological circle, are considered a “hydrological drought”^57^.

In this research, which addresses all types of droughts (i.e., meteorological, agricultural, and hydrological), 1-, 6- and 12-month time scales were used to calculate droughts using the SPEI. Table 1 illustrates the classification of drought severity based on SPEI indices. The thresholds for drought in Table 1 are the most commonly used values in drought studies. They have been used by many researchers for the assessment of droughts based on SPEI all around the world^52,58–61^. These thresholds for drought have also been successfully used in previous studies in the assessment of droughts in Poland^18,21,62^.

**Table 1:** Drought severity classification according to SPEI.

SPEI class	Values
Moderately dry	−1.0 ≥ SPEI > −1.5
Severely dry	−1.5 ≥ SPEI > −2.0
Extremely dry	SPEI ≤ −2.0

The calculation of SPEI values is based on Eqs. 1–3. To calculate the SPEI value as a log-logistic probability distribution, the difference in water balance (WB) is normalized. The following is an equation for expressing the probability density function:

1 $$f\left( x \right)=\frac{\beta }{\alpha }\left( {\frac{{x - \gamma }}{\alpha }} \right){\left[ {1+\left( {\frac{{x - \gamma }}{\alpha }} \right)} \right]^{ - 2}}$$

where the parameters scale is denoted by *α*, the shape is represented by *β*, and the origin is denoted by $\gamma$. As a result, we can express the probability distribution function as follows:

2 $$F\left( x \right)={\left[ {1+{{\left( {\frac{\alpha }{{x - \gamma }}} \right)}^\beta }} \right]^{ - 1}}$$

SPEI is defined^50^ as follows:

3 $${\text{SPEI= W }} - {\text{ }}\frac{{{{\text{C}}_{\text{0}}}{\text{+ }}{{\text{C}}_{\text{1}}}{\text{ W+}}{{\text{C}}_{\text{2}}}{\text{ }}{{\text{W}}^{\text{2}}}}}{{{\text{1 + }}{{\text{d}}_{\text{1}}}{\text{ W + }}{{\text{d}}_{\text{2}}}{\text{ }}{{\text{W}}^{\text{2}}}{\text{ + }}{{\text{d}}_{\text{3}}}{\text{ }}{{\text{W}}^{\text{3}}}}}$$

Whereas, $W=\sqrt { - 2ln\left( P \right)}$when *P* ≤ 0.5, and $W=\sqrt { - 2ln\left( {1 - P} \right)}$ when *P* > 0.5 (where *P* is the probability of exceeding a determined WB value), C0 = 2.5155, C_1_ = 0.8028, C_2_ = 0.0203, d_1_ = 1.4327, d_2_ = 0.1892, d_3_ = 0.0013.

## Results

**Fig. 2:**
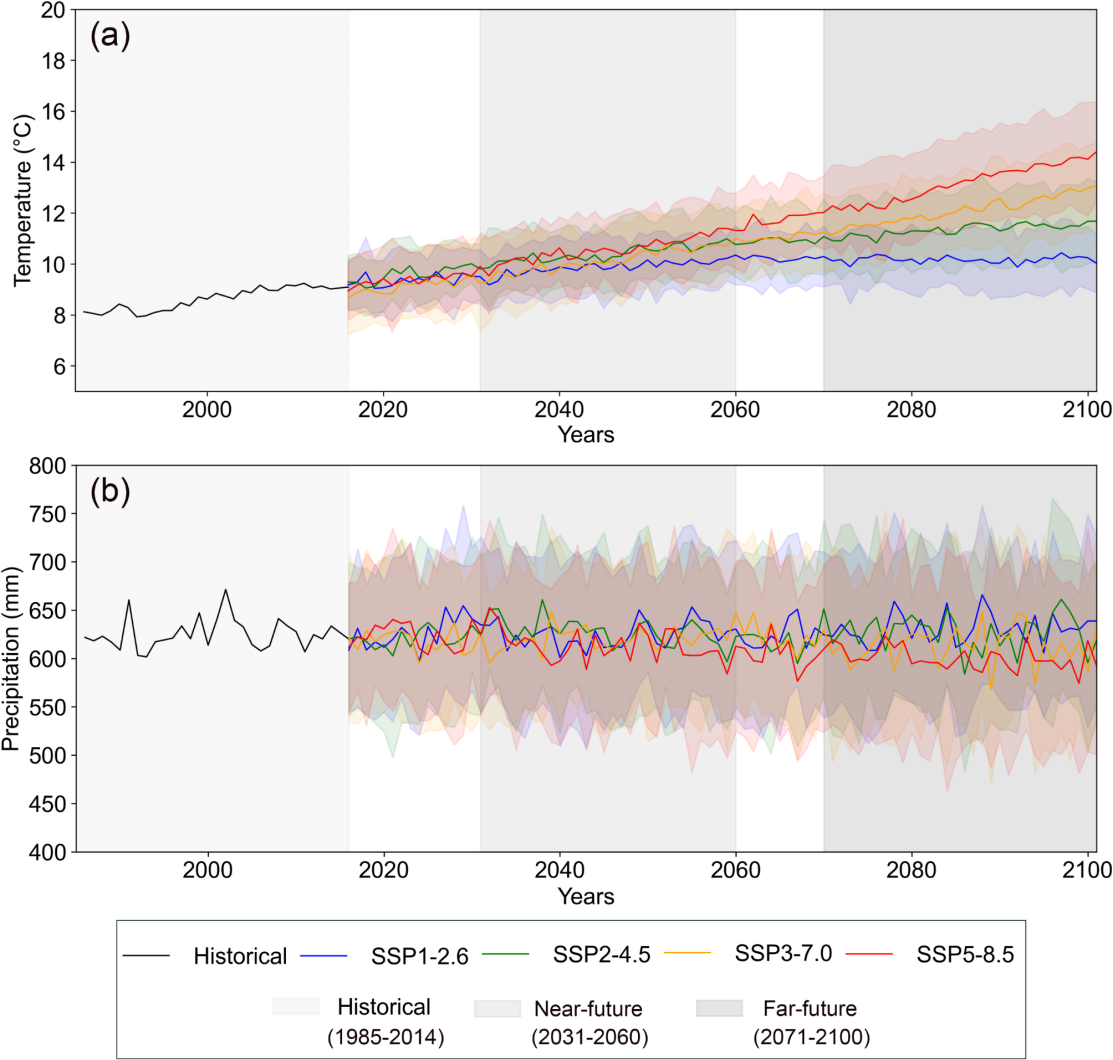
Mean annual temperature (**a**) and annual totals of precipitation (**b**) for the historical and projected future period under SSP scenarios based on multi-model mean of 26-GCMs by the end of the 21st century.

In order to evaluate the impact of climate change on drought frequency and intensity, first, projection of future temperature and precipitation was carried out under SSP scenarios for near-future (2031–2060) and far-future (2071–2100) periods. The time series of temperature and precipitation from the historical reference period by the end of the 21st century is illustrated in Fig. 2.

**Fig. 3:**
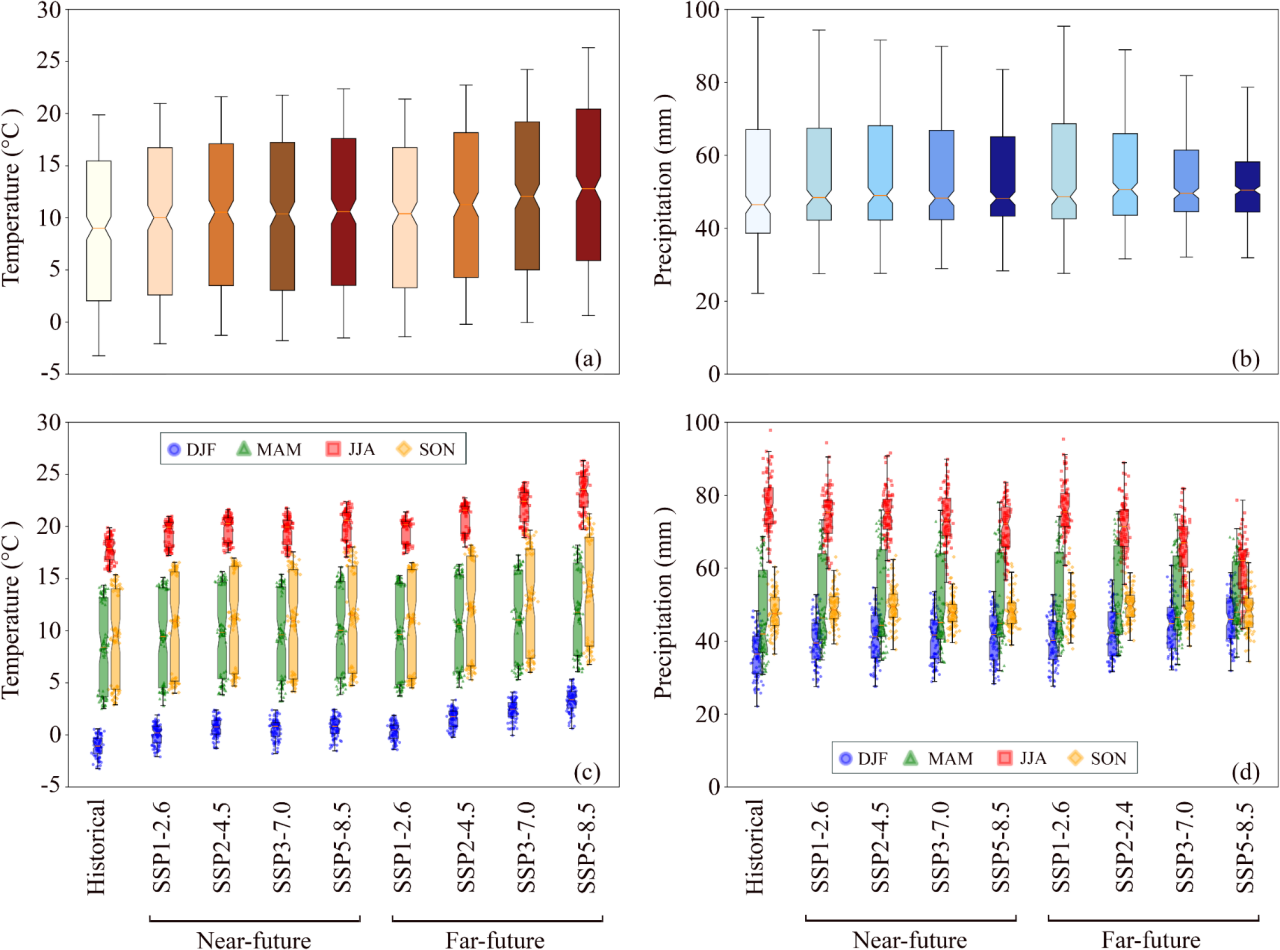
Temperature and precipitation in Poland in historical and future periods: (**a**) average monthly temperature, (**b**) total monthly precipitation, (**c**) average seasonal temperature and (**d**) total seasonal precipitation.

The results presented in Fig. 2 show that temperature will continue to increase in the future (2015–2100) under every SSP scenario. The increases, however, are projected to be significantly greater for the SSP5–8.5 and SSP3–7.0 scenarios than for the SSP1–2.6 and SSP2–4.5 scenarios. Annual temperature (Fig. 2a) of 9.0 °C at the end of the historical period (2014) will rise to 9.95 °C (range: 8.43–11.82 °C), 11.6 °C (range: 9.20–13.44 °C), 12.99 °C (range: 9.89–14.77 °C) and 14.36 °C (range: 10.12–16.36 °C) under SSP1–2.6, SSP2–4.5, SSP3–7.0 and SSP5–8.5, respectively, by the end of the 21st century. Annual precipitation changes are significantly less clear than temperature changes (compare Fig. 2b and a). They show small changes under the SSP1–2.6 and SSP2–4.5 scenarios and even a clear decrease in the case of the SSP3–7.0 and SSP5–8.5 scenarios. In addition to the annual values, we also present mean/totals of monthly and seasonal variations of temperature/precipitation, respectively, for historical and future periods under SSP scenarios (Fig. 3, Fig. S1, and Fig. S2).

Estimation of future changes in the monthly temperature (Fig. 3a and Fig. S1) shows that there is an increase in the average monthly temperature in future periods under all scenarios. In the near-future period, under the SSP1–2.6 scenario, the monthly temperature increases slightly compared to the historical period, while this increase is greater under SSP2–4.5 than under the SSP1–2.6 scenario. The increase in monthly average temperature for SSP3–7.0 and SSP5–8.5 is significantly greater compared to the historical period, SSP1–2.6 and SSP2–4.5 scenarios. Although the average monthly temperature in the far-future period also followed the same trends as the near-future period, the increase in average monthly temperature is dramatic, particularly for the SSP3–7.0 and SSP5–8.5 scenarios. The median temperatures in SSP5–8.5 exceed 15 °C, with maximums reaching ~ 25 °C. The variability also increases significantly, with greater extremes in both colder and warmer months. This suggests a future where extreme heat events could become much more common. Also, the total monthly precipitation changes (Fig. 3b and Fig. S2) in the future period demonstrated that there will be a slight increase in precipitation, particularly under the SSP1–2.6 and SSP2–4.5 scenarios. In contrast, the precipitation will experience a decrease under SSP3–7.0 and SSP5–8.5 scenarios in the far-future period.

Estimation of future changes in seasonal temperature (Fig. 3c) shows that, in all seasons, the temperature increases under all scenarios and follows the same trends for average monthly temperature. The seasonal variations in precipitation (Fig. 3d) reveal that, during the winter (December, January, February), precipitation increases under all scenarios both for near-future and far-future periods, while the trend is downwards for precipitation during the summer (June, July, August).

**Fig. 4:**
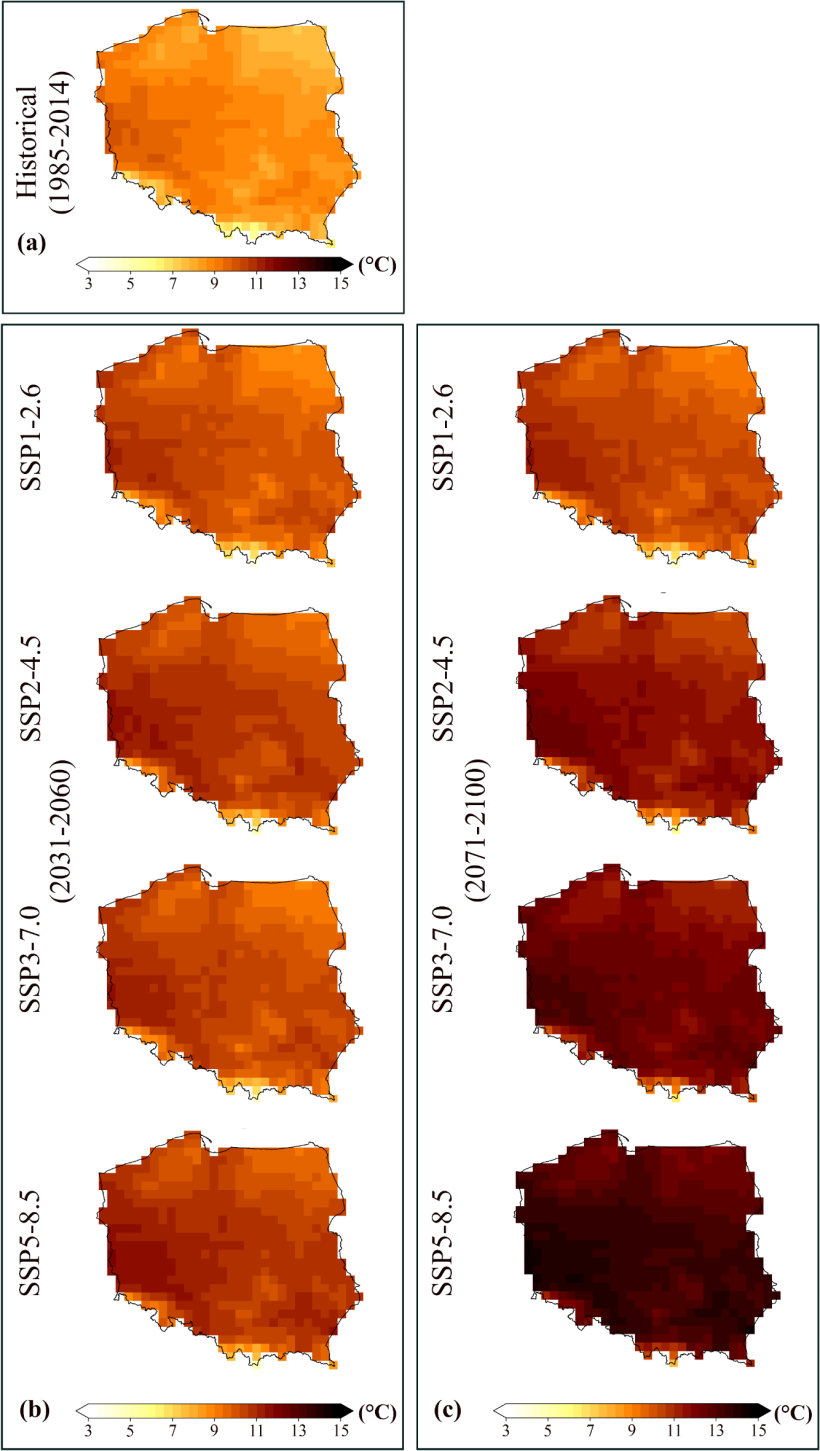
Spatial distribution of annual mean temperature in Poland for (**a**) historical period, (**b**) near-future period, and (**c**) far-future period.

**Fig. 5:**
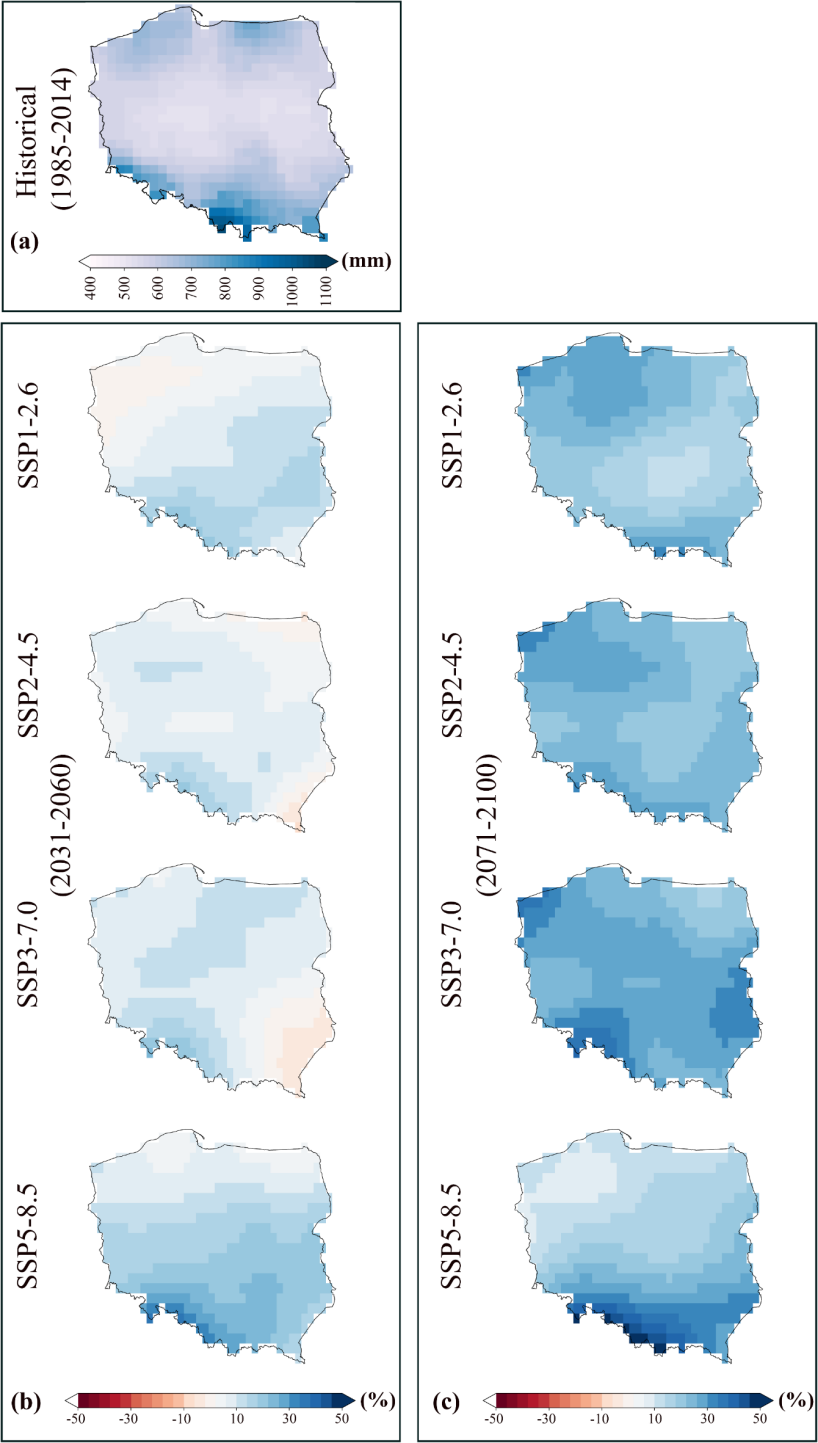
Spatial distribution of annual mean precipitation sums in Poland for (**a**) historical period, (**b**) near-future period, and (**c**) far-future period.

The spatial diversity changes in annual mean temperature and annual mean precipitation sum for the study area for the historical and future are depicted in Figs. 4 and 5. The spatial distribution of temperature changes for the historical period in Poland (Fig. 4a) shows a relatively homogeneous temperature distribution, with temperatures ranging approximately between 5 °C and 11 °C. The northern and eastern regions exhibit slightly lower temperatures than the southwestern regions. In the near-future period (Fig. 4b) under the SSP1–2.6 scenario, the temperature increases slightly, with most regions experiencing temperatures between 7 °C and 11 °C. Under the SSP2–4.5 scenario, temperatures rise to between 9 °C and 13 °C across most areas, with more noticeable warming in the southern parts. In addition, in the near-future, under SSP3–7.0 and SSP5–8.5 scenarios, an increase in temperature would be more significant than in SSP1–2.6 and SSP2–4.5 scenarios, with southern Poland experiencing temperatures exceeding 13 °C, while the northern regions also warm significantly. The spatial changes in temperature for the far-future period under SSP scenarios (Fig. 4c) demonstrated that under SSP1–2.6, temperature increases are still moderate, with temperatures remaining between 7 °C and 11 °C. The temperatures in the SSP2–4.5 scenario continue to rise, with a spatial distribution similar to the near-future but more significant, with southern Poland experiencing temperatures up to 14 °C. In the far-future period and under the SSP3–7.0 and SSP5–8.5 scenarios, the increase in temperature is dramatic and extreme. Under SSP5–8.5, the temperatures across Poland exceed 15 °C, particularly in the southern regions.

The spatial distribution of annual mean precipitation sum in Poland for historical and future periods under SSP scenarios is illustrated in Fig. 5. During the historical period (Fig. 5a), precipitation is greatest in the southern parts of Poland (∼1000 mm) and the north, particularly near the Baltic Coast (700–900 mm). Central regions of Poland receive less precipitation, typically in the range of 500–700 mm. In the near-future (Fig. 5b), most parts of Poland are projected to experience a slight increase (up to 10%) in precipitation. Under the SSP2–4.5 scenario, precipitation also increases slightly by 10%, while there is a minor reduction in precipitation for northern and central regions (around 10%). Under the SSP3–7.0 scenario in the near-future, the precipitation will reduce in southern regions and will slightly increase in northern and central regions. The increase in precipitation under the SSP5–8.5 scenario in the near-future is significant in the southern part (up to 20–30%). The increase in central and northern regions is moderate. In the far-future period under the SSP1–2.6 scenario, the projected precipitation increases are more widespread compared to the near-future period, experiencing increases of 10% and 30%, especially in the northern regions. Under SSP2–4.5, most of Poland is expected to see similar increases in precipitation, ranging from 10 to 30%, particularly in the northern and central regions. For SSP3–7.0, precipitation increases significantly, particularly in the northern and central parts of Poland, with changes ranging from 20 to 50%. In contrast, the southern regions exhibit smaller changes, suggesting a continued pattern of uneven precipitation distribution. Under SSP5–8.5, the most extreme scenario, the largest increases in precipitation are projected, particularly in southern Poland, where changes exceed 30% and even reach 50% in some areas.

**Fig. 6:**
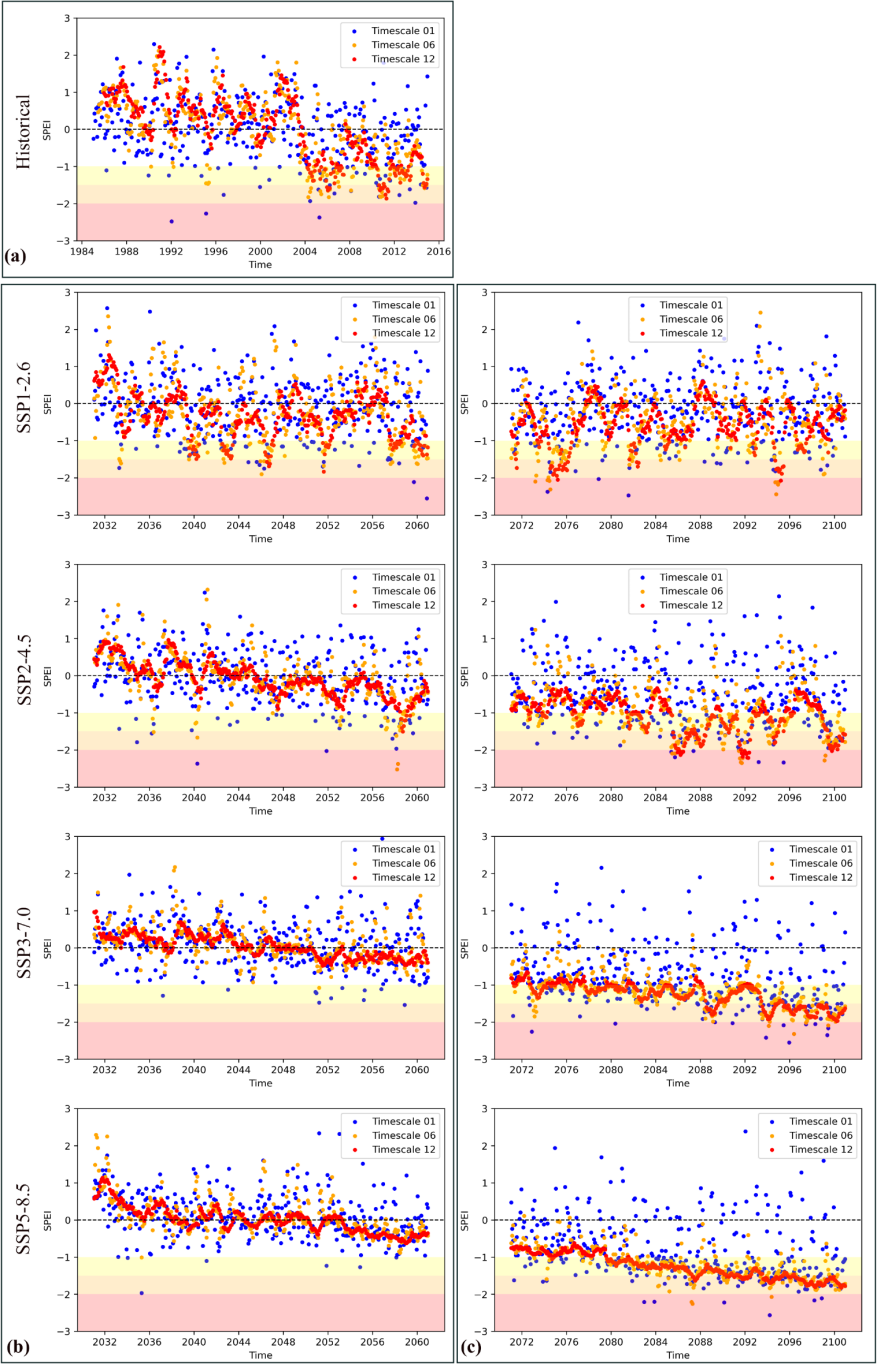
SPEI time series for SPEI 1,6,12 for (**a**) historical, (**b**) near-future and (**c**) far-future periods.

SPEI values were calculated at time scales of 1, 6 and 12 months. The time series of SPEI is depicted in Fig. 6 for historical and future periods with various classes representing moderate, severe and extreme droughts. In the historical period (Fig. 6a), SPEI values fluctuated, with the majority of conditions being near-normal. However, there are periods of moderate and severe droughts, particularly for SPEI-6 and SPEI-12, while extreme drought events are rare. In the near-future period (Fig. 6b), under the SSP1–2.6 and SSP2–4.5 scenarios, the trend shows a similar frequency of moderate droughts across all timescales, with a relatively small number of severe droughts and rare extreme droughts. In the far-future period, generally, the frequency of droughts will increase under all SSP scenarios. Under SSP3–7.0 and SSP5–8.5 scenarios, the frequency of moderate and severe droughts will increase significantly, particularly for SPEI-6 and SPEI-12 scales, while there is no noticeable increase in extreme droughts for future periods.

**Fig. 7:**
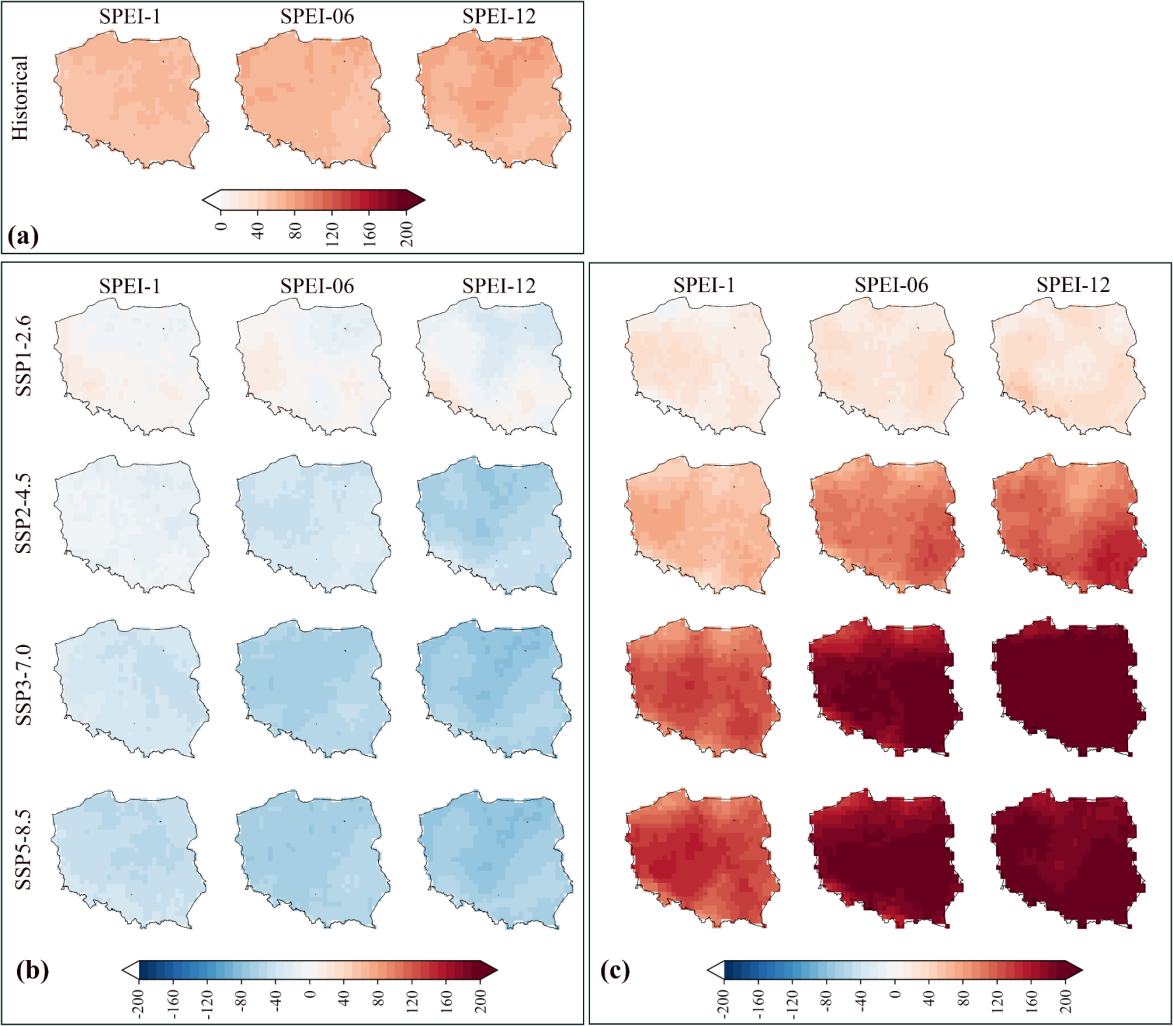
Frequency of droughts in Poland in the historical period (**a**) and its changes in the near-future (**b**) and far-future (**c**). Differences are shown relative to the historical period.

In addition to the time series of droughts, spatial changes in the frequency of droughts are presented in Fig. 7 (total frequency for all drought classes) and Figs. S3–S5 (frequency of severe, moderate and extreme droughts). The frequency of all drought types (moderate, severe and extreme) presented in Fig. 7 shows that, in the near-future period, there will be a decreasing trend in the frequency of droughts. In contrast, in the far-future period, the frequency of droughts will increase significantly under all scenarios, excluding the SSP1–2.6 scenario, for which the rise will be negligible. The increased frequency of droughts under SSP3–7.0 and SSP5–8.5 scenarios will be dramatic, particularly for the SPEI-6 and SPEI-12 scales.

The spatial changes in the frequency of moderate and extreme droughts (Figs. S3 and S5) exhibited a similar pattern as the total frequency of droughts, with a decrease in the frequency of droughts in the near-future period and an increase under SSP2–4.5, SSP3–7.0 and SSP5–8.5 scenarios. The frequency of severe droughts will increase significantly under SSP3 − 7.0 and SSP5 − 8.5 scenarios (Fig. S4). By evaluating the frequency of extreme droughts (Fig. S5), it was revealed that the frequency of extreme droughts will increase for SPEI-6 and SPEI-12 under the SSP1 − 2.6 scenario, all time scales under the SSP2–4.5 scenario and only SPEI-1 and SPEI-6 under SSP3–7.0 scenario. The frequency of extreme droughts under the SSP5–8.5 scenario is similar as in the historical period. To summarize, the changes in the frequency of droughts for the study area in the historical and future periods are depicted in Fig. S6.

## Discussion

Assessment of future climate conditions and their impact depends on the quality of available datasets. Although regional climate models (RCMs) perform well compared to the GCMs in the evaluation of future climate, the development of such a larger ensemble of RCMs by various research groups, such as the Coordinated Regional Downscaling Experiment (CORDEX), takes several years after the release of GCMs. To date, there are no such regional models available for the study area. Thus, a set of high-resolution downscaled and biased-corrected models, which have been approved in many studies, was used to project future climate conditions and assess occurrences of droughts in Poland.

To discuss the results in detail, this study’s output was compared with the results of available studies projecting temperature, precipitation and occurrences of droughts in Poland.

The results in the projection of temperature for the area of Poland in this study showed that temperature will increase by 1–4.5 °C under SSP scenarios by the end of the 21st century compared to the historical reference period. The increase in the average temperature of the near-future period relative to the reference period is in the range 1–1.95 °C, while it will reach 1.5–4.5 °C for the far-future period. Mezghani, et al.^22^, by developing high-resolution climate models for Poland based on the CMIP5 dataset under RCP scenarios, estimated an increase of 1–2 °C in temperature for the near-future under RCP2.6 and RCP4.5 scenarios and 4 °C for the far-future (2071–2100) under RCP8.5. Piniewski, et al.^63^ focused on the two main river basins of Poland (the Vistula and Oder river basins), estimating temperature increases of 1–1.5 °C for the near-future and 1.8–3.7 °C for the far-future. Szwed^25^ similarly projected temperature rises for Poland, with 1 °C (RCP4.5) and 1.5 °C (RCP8.5) increases over the reference period in the case of 2021–2050 and 2 °C (RCP4.5) and 3–4 °C (RCP8.5) for 2071–2100.

The difference in the capturing of temperature increase between this study and the mentioned studies may relate to the inherent differences between CMIP5 and CMIP6 models and scenarios. Studies focusing on the comparison of CMIP5 and CMIP6 model results have stated that CMIP6 models improved performance in simulating temperature over the CMIP5 ^64–66^. The main reason behind this fact is related to the sensitivity of CMIP6 models and shifts in greenhouse gas concentrations from CMIP5 to CMIP6 ^67^. In addition, Fredriksen, et al.^68^, by comparing the mentioned models, concluded that CMIP6 models generally exhibit higher sensitivity due to the fact that forcing estimates for the 21st century increase more rapidly in CMIP6 compared to CMIP5, which is attributed to higher CO_2_ concentrations in future CMIP6 scenarios. This could justify the greater temperature rise projected in future scenarios under CMIP6. There are several studies that have confirmed that the increase in temperature in CMIP6 is higher than that for CMIP5 ^67,69^. This fact is also confirmed by studies for projecting temperature change in Poland under SSP scenarios from CMIP6 models^26,38^.

In contrast to the temperature, precipitation in future periods in Poland fluctuates depending on climate scenarios, period and seasons. In the near-future, precipitation will increase based on the results achieved in this study, which also corresponds with the available literature. Kundzewicz, et al.^17^, based on CMIP5 models, stated that annual precipitation in Poland will increase by 6–10% in the near-future and 8–16% in the far-future period. Kęsicka, et al.^70^ projected an increase of 7.5% in annual precipitation under SSP3–7.0 for Poznań, Poland. Another interesting result is decreasing summer precipitation under all scenarios in both future periods. These results were also highlighted in previous studies globally^12,71,72^ and in European climate projections^73–75^. For example, Palmer, et al.^76^ concluded that summer precipitation will reduce in CMIP6 for Central Europe. Holtanová, et al.^77^ projected mean changes in temperature and precipitation over the Czech Republic and concluded that monthly mean precipitation is decreasing for Central Europe.

The calculated SPEI values for the historical period show that periods of moderate and severe droughts are increasing, especially for SPEI-6 and SPEI-12, for this period, whereas extreme drought events remain rare. These results are also highlighted in previous studies focusing on drought events by SPEI for various time periods^62,78,79^.

The projected change in frequency and intensity of droughts based on SPEI under SSP scenarios showed that the frequency of droughts in the near-future decreases, while there are increasing trends for drought frequency under SSP2–4.5, SSP3–7.0 and SSP5–8.5 scenarios in the far-future period. These results were also stated in the study by Meresa, et al.^21^, who concluded that the projected droughts will only increase in the far-future based on SPEI. In general, according to the findings of this study, two classes of severe and moderate droughts will experience an increase in the frequency for the far-future period, while the frequency of extreme droughts increases only for SPEI-6 and SPEI-12 under the SSP1–2.6 scenario, SPEI-1,6,12 under the SSP2–4.5 scenario and under SPEI-1 and SPEI-6 under the SSP3–7.0 scenario. The results demonstrated a dramatic increase in agricultural and hydrological droughts (SPEI-6 and SPEI-12) in Poland, particularly under SSP3–7.0 and SSP5–8.5 scenarios in the far-future period. The increase in frequency and severity of droughts in Poland corresponds relatively well with studies focusing on the future of droughts in Europe and Poland^21,27,80^. In the latest study for Poland by Marcinkowski et al.^81^, the authors evaluated droughts in Poland based on the calculation of different methods for PET and various drought indices from CMIP5 under the RCP8.5 scenario. The authors concluded that from 2020 to 2050 droughts will slightly increase, while the increase in the duration of droughts in the period of 2050–2080 will be more than in previous periods. Thus, despite the differences in the time period, climate models and scenarios, the finding of the mentioned study is also aligned (with only slight differences) with our results. In addition, some studies, such as Spinoni et al.^82^, also highlighted that despite the increase in drought severity (such as an extreme type) in Europe, there are some exceptions, such as Poland, Hungary, Switzerland, Lithuania and Belarus (see the conclusion and summary section in the mentioned paper). Therefore, based on the findings of this study, this fact may partially be related to a decrease in extreme droughts in Poland in the future period.

In conclusion, we would like to highlight that projections of future climate conditions and extreme events such as droughts inherently include uncertainties. The main sources of these uncertainties include the selection of GCM models, climate change scenarios, time periods, and quality of datasets. Given these uncertainties, it is possible that the results of this study may differ slightly from those of other studies.

To summarize, there is no doubt that human-induced climate change will cause various catastrophic events, such as droughts, in the future. Increased frequency of agricultural and hydrological droughts in Poland will pose significant risks to future water and food security, particularly in far-future periods.

## Conclusions

This research provides a comprehensive projection of changes in temperature and precipitation, as well as droughts, for the future in Poland. To this end, an average mean of 26 models from CMIP6 was used under four common and complete scenarios (SSP1–2.6, SSP2–4.5, SSP3–7.0 and SSP5–8.5) for two future periods: near-future (2031–2060) and far-future (2071–2100).

To conclude, the results indicated that temperature in Poland will increase by 1–4.5 °C by the end of the 21st century, with significant increases projected under SSP3–7.0 and SSP5–8.5 scenarios. In addition, there will be a slight increase in precipitation in Poland, excluding the SSP5–8.5 scenario in the near-future. The increase in temperature, coupled with variations in precipitation, causes the frequency and intensity of droughts to increase for the far-future period, whereas it will decrease for the near-future period.

The findings of this study offer valuable insights for the scientific community in assessing the potential impacts of climate change on extreme hydrological events. The significant increase in temperature and projected precipitation declines in summer highlight potential vulnerabilities in crop yields and water availability. Moreover, according to the results, increasing the frequency of agricultural and hydrological droughts in Poland provides critical information for decision-makers to develop strategies and measures for water and food security, particularly in far-future periods.

## Electronic supplementary material

Below is the link to the electronic supplementary material.

Supplementary Material 1

## Data Availability

All datasets of climate variables (i.e., temperature and precipitation) for GCMs from CMIP6 used for this study are publicly available at https://registry.opendata.aws/nex-gddp-cmip6 (accessed on 20 September 2023) and from the NEX-GDDP-CMIP6 NASA Center for Climate Simulation. The additional data that support the findings of this study are available from the corresponding author upon reasonable request.
