# Supplementary material for: Projection of climate change impact on the occurrence of drought events in Poland
Source: Sci Rep. 2025 Feb 15;15:5609. doi: 10.1038/s41598-025-90488-0 (PMC11829970; doi:10.1038/s41598-025-90488-0)
Supplement: Supplementary file 1 — Supplementary Material 1 [file 41598_2025_90488_MOESM1_ESM.pdf]

Scientific Reports

Supplementary material for "Projection of climate change impact on the occurrence of drought events in Poland"

Babak Ghazi [a], Hossein Salehi [b], Rajmund Przybylak [a, c], Aleksandra Pospieszyńska [a, c]

[a] Department of Meteorology and Climatology, Faculty of Earth Sciences and Spatial Management, Nicolaus Copernicus University, Toruń, Poland

[b] Department of Physics, University of Trento, 38123 Trento, Italy

[c] Centre for Climate Change Research, Nicolaus Copernicus University, Toruń, Poland

* Corresponding author: Babak Ghazi, babak.ghazi@doktorant.umk.pl

Table S1. List of employed GCMs for multi-model mean ensemble

01	ACCESS-CM2
02	ACCESS-ESM1-5
03	BCC-CSM2-MR
04	CanESM5
05	CESM2
06	CMCC-CM2-SR5
07	CMCC-ESM2
08	CNRM-CM6-1
09	CNRM-ESM2-1
10	EC-Earth3-Veg-LR
11	EC-Earth3
12	FGOALS-g3
13	GFDL-ESM4
14	GISS-E2-1-G
15	INM-CM4-8
16	INM-CM5-0
17	IPSL-CM6A-LR
18	KACE-1-0-G
19	MIROC6
20	MPI-ESM1-2-HR
21	MPI-ESM1-2-LR
22	MRI-ESM2-0
23	NorESM2-LM
24	NorESM2-MM
25	TaiESM1
26	UKESM1-0-LL

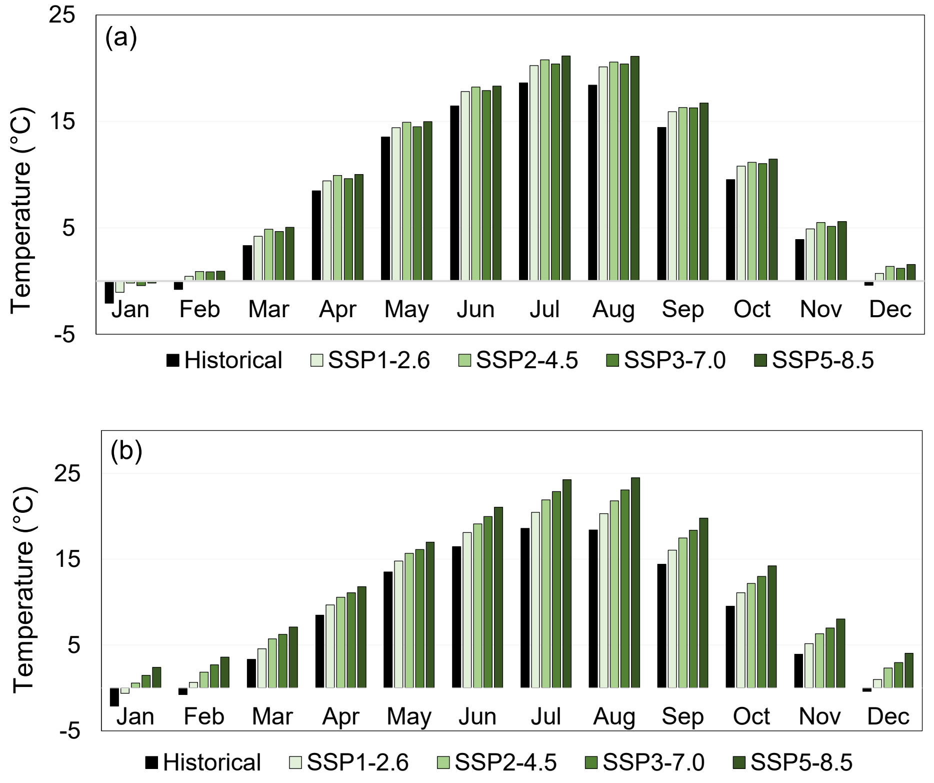

Fig. S1. Monthly variations of temperature for the historical period and projected future periods (a) 2031–2060 (b) 2071–2100, under SSP scenarios

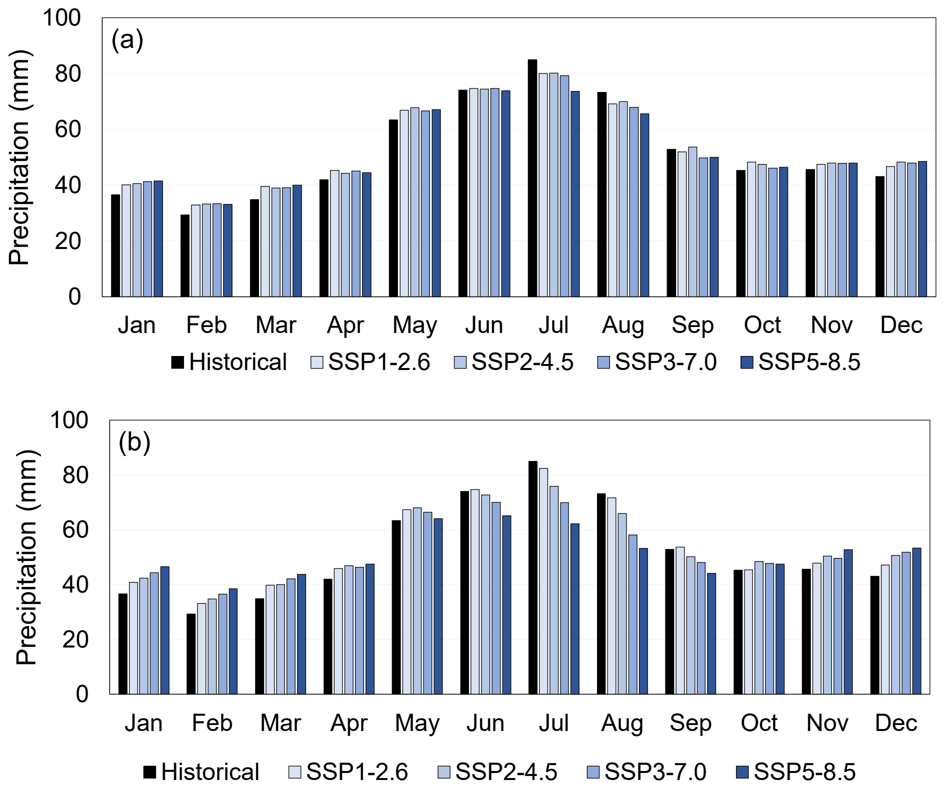

Fig. S2. Monthly variations of precipitation for the historical period and projected future periods (a) 2031–2060 (b) 2071–2100, under SSP scenarios

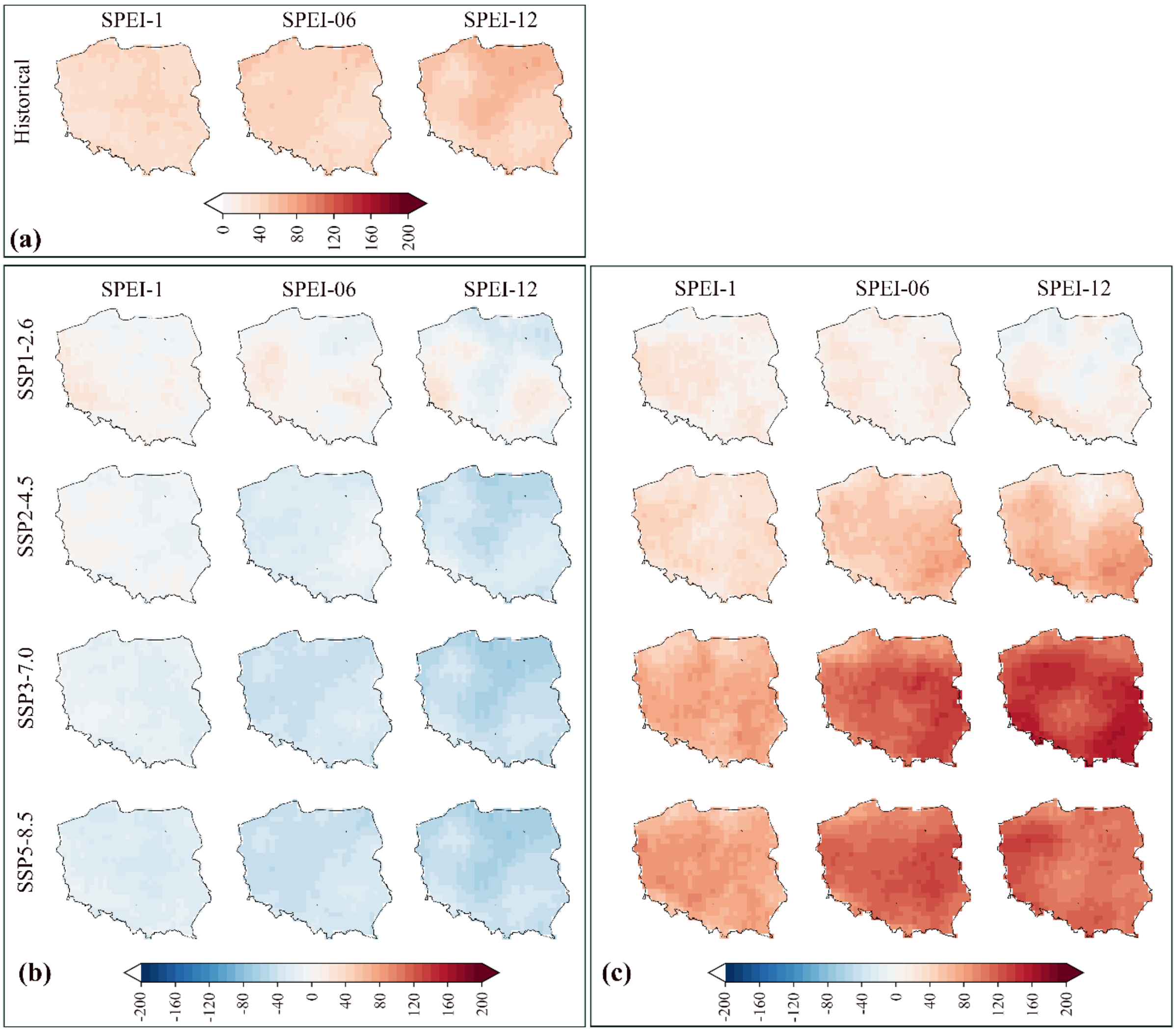

Fig S3. The frequency of moderate droughts in Poland in (a) historical period and its changes in the (b) near-future period and (c) far-future period. Differences are shown relative to the historical period.

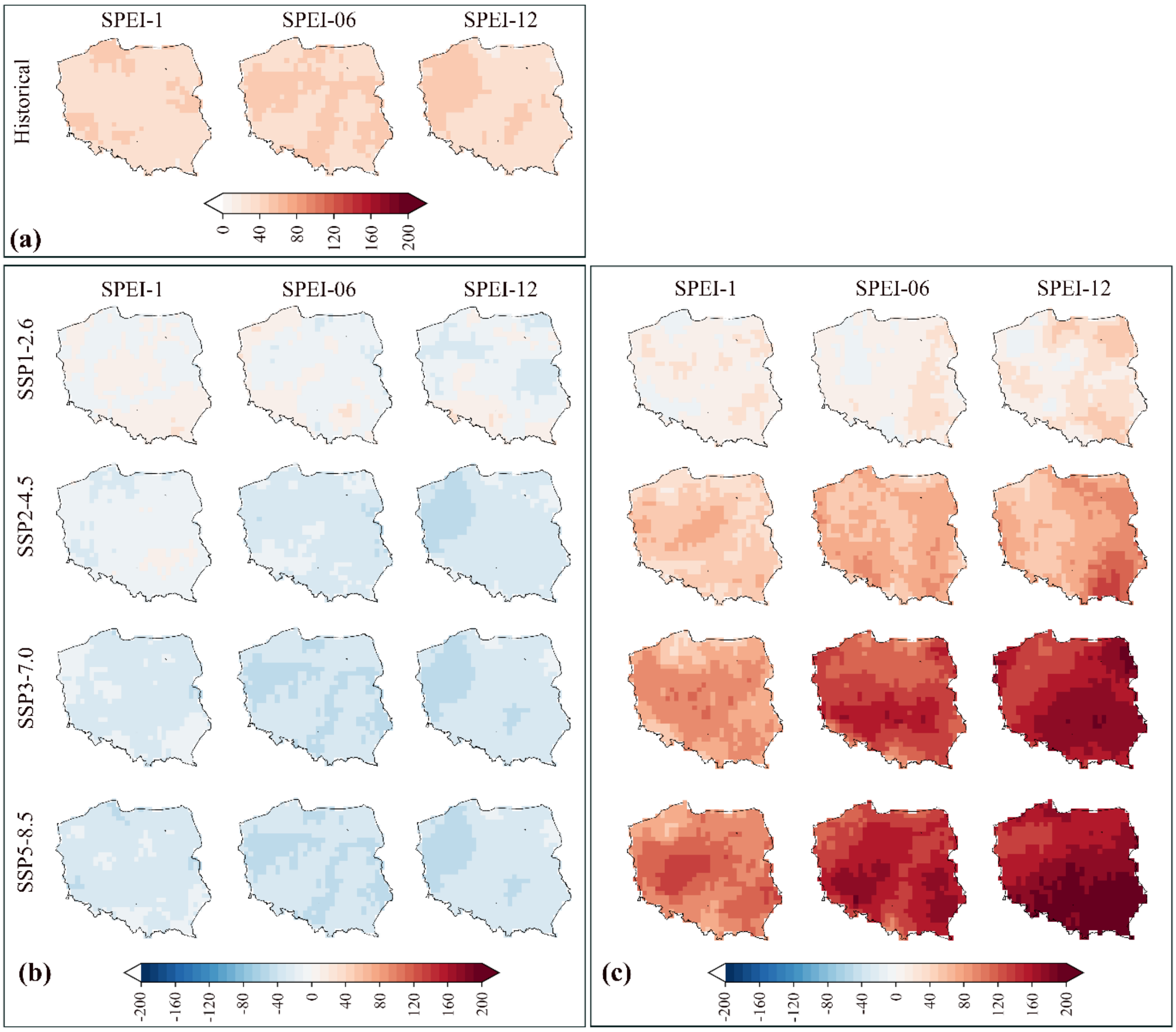

Fig S4. The frequency of severe droughts in Poland in (a) historical period and its changes in the (b) near-future period, and (c) far-future period. Differences are shown relative to the historical period.

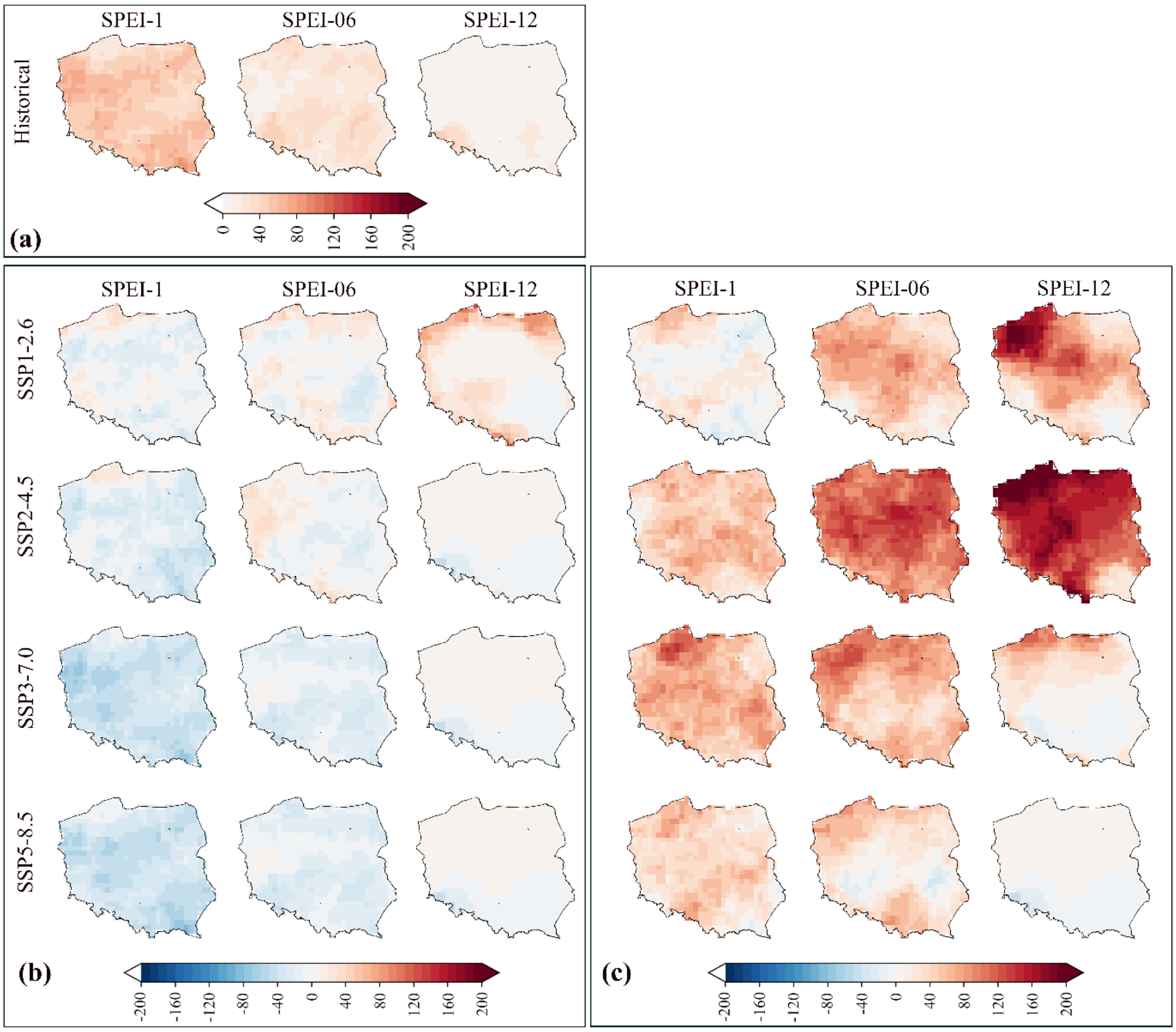

Fig S5. The frequency of extreme droughts in Poland in (a) historical period and its changes in the (b) near-future period and (c) far-future period. Differences are shown relative to the historical period.

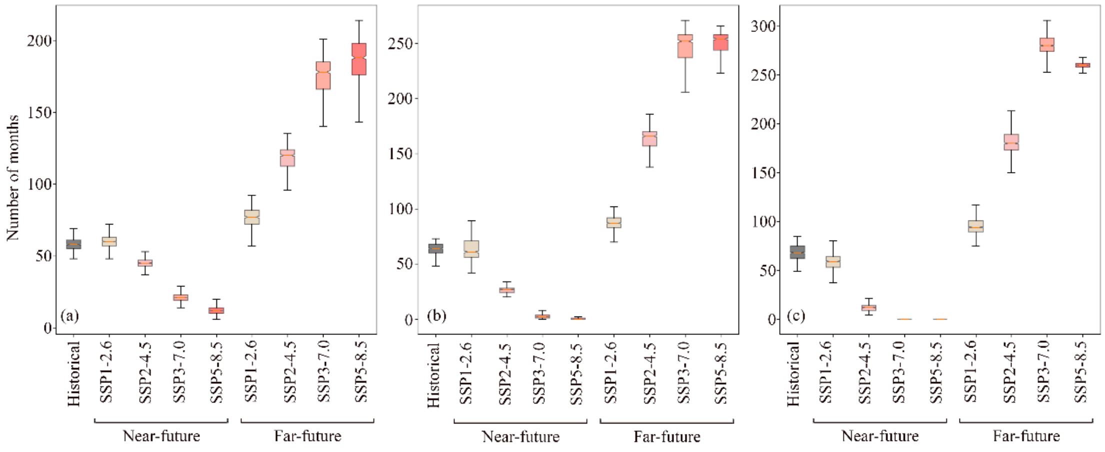

Fig. S6. Change in the frequency of droughts in historical and future periods under SSP scenarios for (a) moderate droughts, (b) severe droughts, and (c) extreme droughts
